# Apolipoprotein A4 Defines the Villus-Crypt Border in Duodenal Specimens for Celiac Disease Morphometry

**DOI:** 10.3389/fimmu.2021.713854

**Published:** 2021-07-29

**Authors:** Juha Taavela, Keijo Viiri, Anna Välimäki, Jani Sarin, Kristiina Salonoja, Markku Mäki, Jorma Isola

**Affiliations:** ^1^Central Finland Central Hospital, Jyväskylä, Finland; ^2^Celiac Disease Research Center, Faculty of Medicine and Health Technology, Tampere University, Tampere, Finland; ^3^Fimlab Laboratories Inc, Tampere, Finland; ^4^Jilab Inc, Tampere, Finland; ^5^Tampere University Hospital, Tampere, Finland; ^6^Faculty of Medicine and Health Technology, Tampere University, Tampere, Finland

**Keywords:** celiac disease, morphometry, duodenal biopsy, histology, gluten challenge, apolipoprotein A4, digital pathology, clinical trial

## Abstract

Histological evaluation of the small intestinal mucosa is the cornerstone of celiac disease diagnostics and an important outcome in scientific studies. Gluten-dependent injury can be evaluated either with quantitative morphometry or grouped classifications. A drawback of mucosal readings is the subjective assessment of the border where the crypt epithelium changes to the differentiated villus epithelium. We studied potential immunohistochemical markers for the detection of the villus-crypt border: apolipoprotein A4 (APOA4), Ki-67, glucose transporter 2, keratin 20, cytochrome P450 3A4 and intestinal fatty-acid binding protein. Among these, villus-specific APOA4 was chosen as the best candidate for further studies. Hematoxylin-eosin (H&E)- and APOA4 stained duodenal biopsy specimens from 74 adult patients were evaluated by five observers to determine the villus-to-crypt ratio (VH : CrD). APOA4 delineated the villus to crypt epithelium transition clearly, and the correlation coefficient of VH : CrD values between APOA4 and H&E was excellent (r=0.962). The VH : CrD values were lower in APOA4 staining (p<0.001) and a conversion factor of 0.2 in VH : CrD measurements was observed to make the two methods comparable to each other. In the intraobserver analysis, the doubled standard deviations, representing the error ranges, were 0.528 for H&E and 0.388 for APOA4 staining, and the ICCs were 0.980 and 0.971, respectively. In the interobserver analysis, the average error ranges were 1.017 for H&E and 0.847 for APOA4 staining, and the ICCs were better for APOA4 than for H&E staining in all analyses. In conclusion, the reliability and reproducibility of morphometrical VH : CrD readings are improved with the use of APOA4 staining.

## Introduction

Celiac disease is an autoimmune disorder in which dietary gluten causes an immunological reaction manifesting as gradual development of small bowel mucosal damage (1). Small bowel damage consists of sequential and slow development of lymphocytosis, crypt hyperplasia and villus atrophy (2). Currently, the only treatment for celiac disease is a life-long gluten-free diet. However, dietary management is not sufficient for many patients with celiac disease, and up to 40% of patients suffer from symptoms even on this diet (3). Additionally, the duodenal mucosa may not heal sufficiently on this diet, causing risks of complications and micronutrient deficiencies (4, 5). Interestingly, there are several ongoing gluten challenge studies assessing the efficacy of candidate drugs and vaccines for celiac disease (6). In these studies, it is of utmost importance to ensure that the drug, device, or vaccine protects against mucosal damage, as it is the only marker that is linked to the long-term health of the patient, risk of complications, and mortality (7–9).

Mucosal damage can be evaluated histologically with either categorical classifications or quantitative measurements. Categorical classifications such as the Marsh-Oberhuber and Corazza-Villanacci classifications are the most commonly used in routine clinical practice because of their ease of use (1, 10). These classifications combine the parameters of duodenal damage, intraepithelial lymphocyte (IEL) density, crypt depth (CrD) and villus height (VH) into a single class describing the level of mucosal damage. A more detailed analysis can be performed with the use of quantitative measurements such as the villus height-to-crypt depth ratio (VH : CrD) and density of CD3-positive IELs, which allow the detection of small but significant changes that are not detectable with categorical variables (11–13). Hence, it is preferred to use these continuous mucosal readouts separately for morphology and inflammation in rigorous scientific studies, such as in celiac disease drug/device/vaccine trials (12, 14).

**Figure 1:**
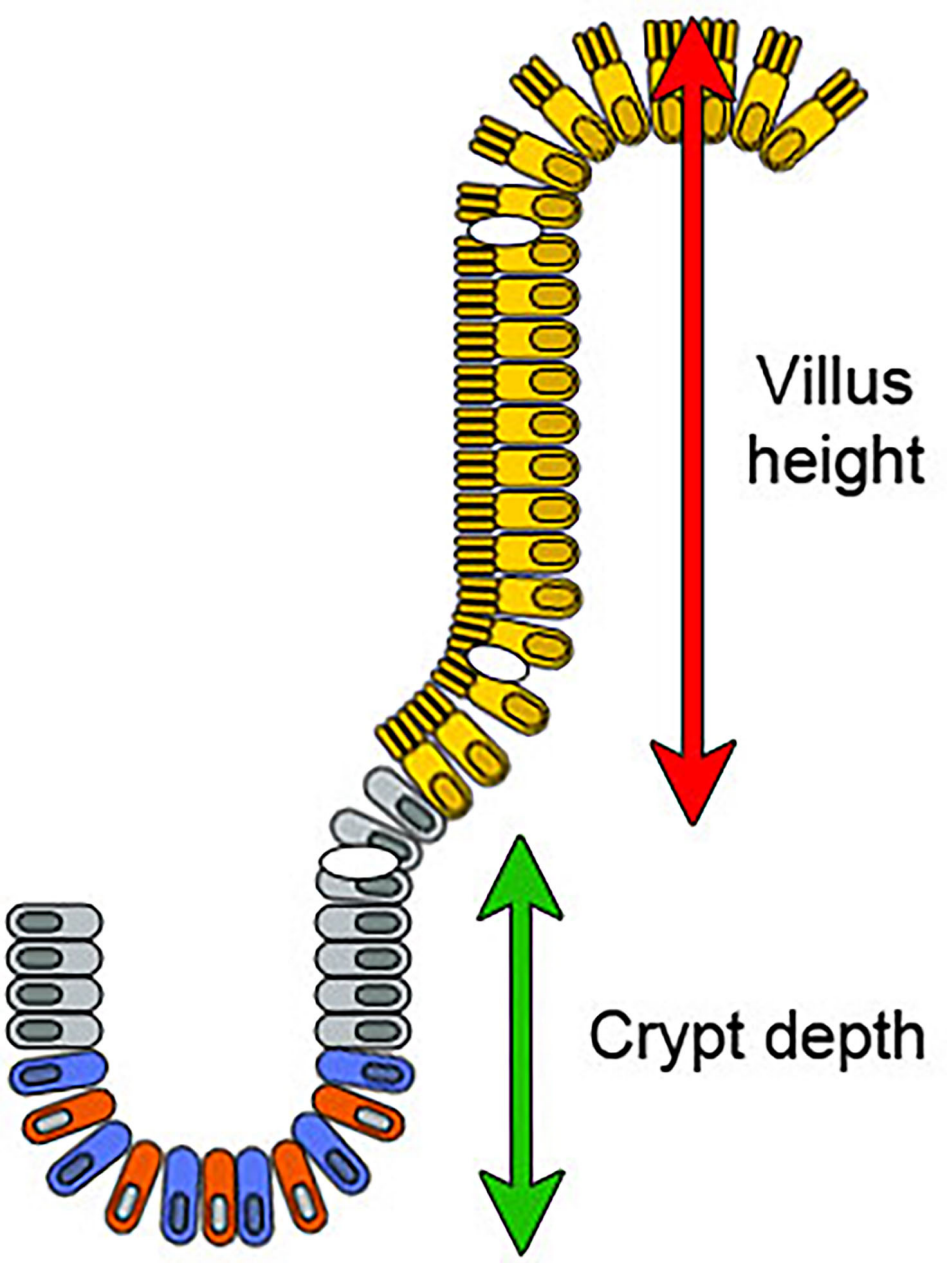
A diagram of the intestinal epithelium in the villus-crypt axis. The crypt generates new cells that differentiate and migrate towards the tip of the villus. The crypt base columnar cells (blue) divide continuously and function as intestinal stem cells. Paneth cells (red) are also at the crypt bottom and nurse these stem cells. Above the stem cell zone is the zone of transit amplifying cells containing lineage-committed progenitor cells (gray). Fully mature absorptive epithelial cells displaying organized microvilli (villus enterocytes, in yellow) emerge from the crypt and move towards the villus tip. Goblet cells are present in both the crypts and villi (shown in white). Enteroendocrine cells are localized among mature enterocytes (not shown). The green and red arrows show villus height and crypt depth measurements in the model.

Recent studies have shown poor reliability and reproducibility when using the results of grouped classifications in assessing duodenal specimens (15–19). There are several pitfalls in the assessment of duodenal biopsy specimens that explain these difficulties (11, 16, 17). An incorrect (tangential) cutting plane of the biopsy is currently a well-established source of error (11), but another fundamental problem is the definition of the border between differentiated villus enterocytes and the proliferating crypt epithelium (20). The distinction between small bowel villi and crypt epithelium can be made by the presence of fully differentiated microvilli revealed only by electron microscopy (**Figure 1**) (21). To date, specific markers for the villus-crypt border to be used in light microscopy have not been identified. Currently, the use of standard hematoxylin-eosin (H&E) staining makes it difficult to define exactly the epithelial transition zone determining the villus-crypt border. Understandably, as readers use their own experience in the assessment of the villus-crypt border, the results between readers have shown high interobserver variability (11, 16, 17, 19). The histopathological diagnosis (celiac disease vs normal) has even changed in up to 11% of cases when the samples have been reread (15). Even a small variation in the point where villus ends and crypt begins is multiplied when calculating the VH : CrD ratio, as it consists of two mutually dependent measurements (VH and CrD). Therefore, it would be of significant benefit to develop an objective marker of the villus-crypt border that would harmonize celiac disease diagnostics and increase measurement reliability and reproducibility. Hence, we studied several potential proteins to find an immunohistochemical marker that would define the exact border between villi and crypts.

## Materials and Methods

### Patients and Biopsies

The material comprised 74 small intestinal mucosal specimens from 74 patients, which were obtained from a prospectively collected database and biobank maintained by our study group. Altogether, 6 specimens were obtained from newly diagnosed untreated celiac patients, 6 from patients on a gluten-free diet, 32 from patients who underwent gluten challenge (22) and 30 specimens from nonceliac controls. The mean age of the celiac patients was 57 years (range 15–63), and 63% were women. The mean age of the nonceliac controls was 57 years (range 17–86), and 52% of them were women. Small bowel biopsies were selected to represent variable stages of mucosal injury ranging from completely normal histology to overt mucosal atrophy and crypt hyperplasia. According to Marsh-Oberhuber grading (23), duodenal injury in the specimens was Marsh 0 (n=15), Marsh 1 (n=10), Marsh 2 (n=10), Marsh 3a (n=13), Marsh 3b (n=12) and Marsh 3c (n=9).

The forceps biopsy specimens were formalin-fixed and embedded in paraffin wax according to standard pathology practice. Standard 3- to 4-µm-thick sections were cut under a microscope to achieve the correct orientation and were then stained with hematoxylin and eosin. Slides were scanned as high-resolution whole slide images at a resolution of 0.17 µm per pixel (SlideStrider scanner, Jilab Inc., Tampere, Finland). Additional sections were cut and used for immunohistochemical (IHC) experiments. Figure panels and art work were created with Adobe Photoshop CS5 (Adobe Inc., CA, USA).

### APOA4, Ki-67, GLUT2, KRT20, CYP3A4 and I-FABP Immunohistochemistry

We surveyed the existing genome-wide studies (4, 24) and the Human Protein Atlas (25) to identify candidate IHC markers that would preferentially label villi or crypt epithelium to define the villus-crypt border exactly. The most promising candidate proteins—apolipoprotein A4 (APOA4), antigen KI-67 (Ki-67), glucose transporter 2 (GLUT2), keratin-20 (KRT20), cytochrome P450 3A4 (CYP3A4) and intestinal fatty-acid binding protein (I-FABP)—were selected for preliminary staining experiments. The antibodies and their working dilutions are described in **Supplementary Table 1**. For all antibodies, a standard IHC staining protocol using high pH, heat-induced antigen retrieval (incubation at 121°C for 2 min in 0.01 Tris-EDTA buffer, pH 9.0), blocking of endogenous peroxidase activity (3% H_2_O_2_ for 5 min at RT), and a 60-min incubation with primary antibodies (60 min at RT) were used. Bound antibodies were visualized with anti-mouse/anti-rabbit peroxidase polymer and DAB chromogen (HistoFine kit, Nichirei Biosciences, Nichirei, Japan). Slides were counterstained with hematoxylin and mounted with DPX (Sigma-Aldrich, MO, USA). Immunohistochemical staining was carried out with an automated IHC-staining device (LabVision Autostainer; Thermo Fisher, Waltham, MA, USA). Slides were scanned as whole slide images.

After the selection of APOA4 for further analysis, the previously H&E stained and analyzed slides were soaked in xylene for up to 3-4 days to dissolve the mounting medium and to detach the coverslips. Slides were then rehydrated and stained with APOA4 IHC as described above. The polyclonal APOA4 antibody was used for the stainings because its use is well documented and found to be rather specific for duodenum (25). The staining was also tested with monoclonal APOA4 antibody and its staining pattern appeared to be similar to that of the polyclonal antibody (not shown). Eosin was added to the counterstain to visualize the Paneth cells at the crypt bottom.

### Digital Measurement of VH and CrD

All IHC-stained slides were scanned as whole-slide images as described above. The sections were viewed and analyzed with web-based client software (Celiac Slide Analyzer) according to our standard operating procedure (11, 24). The small intestinal mucosal VH : CrD was evaluated in all measurable (at least three) separate villus-crypt units, and the result was given as the average of the ratios. VH and CrD were measured digitally by drawing segmented lines whose lengths were calibrated to micrometers (24). Only well-oriented villus-crypt units in the samples, ie. perpendicular to the luminal surface, were allowed to be assessed.

Five academic observers 1, 2, 3, 4 and 5 (JT, JS, KS, AV, JI) analyzed all slides in a blinded fashion independently and were unaware of the clinical data or laboratory findings of the patients. Additionally, one evaluator evaluated the specimens twice with 1 month between the measurements (JT). The villus crypt units identified and measured on the H&E image were relocalized on the APOA4-IHC whole slide image. In the APOA4-stained specimens (digital images), VH : CrD measurements were performed using APOA4 labeling to define the border of the villus and crypt.

### Statistics

Intraobserver and interobserver variations were analyzed by the Bland-Altman method, linear regression analyses, and intraclass correlation coefficients (ICCs) (26, 27). In the Bland-Altman method, the differences between two quantitative measurements are plotted against the averages of the two measurements, and the results are reported as the mean difference between the two measurements and limits of agreement, which are defined as the mean difference plus and minus twice the standard deviation of the differences. In the Bland-Altman plot, the x-axis shows the mean of the results of the two measurements, and the y-axis represents the absolute difference between the two measurements. The intraobserver, interobserver and intermethod agreement was assessed with ICC, and intermethod correlations were assessed by Pearson correlation analysis. Correlation coefficients were considered excellent (above 0.9), strong (0.7-0.9), moderate (0.4–0.6), weak (0.1–0.4) or negligible (0.0-0.1) (28). Quantitative data are expressed as the number of subjects (n), mean and ranges. A paired samples t-test was used to compare the means between groups.

## Results

**Figure 2:**
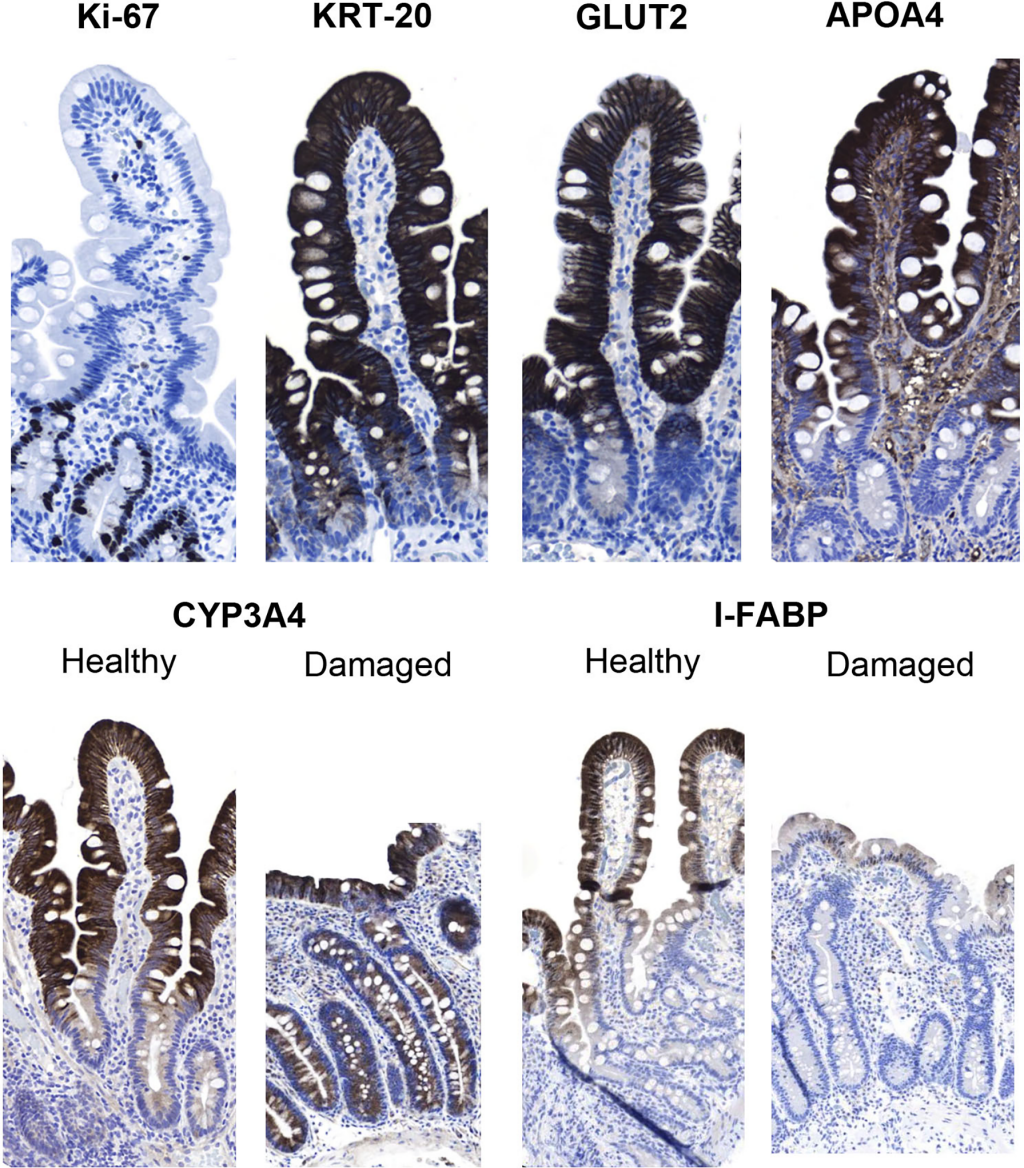
Immunohistochemical analysis of the potential markers of the villus-crypt border in duodenal biopsy specimens. Ki-67 labels the crypt cells, but the labeling does not extend up to the villus-crypt border. Keratin 20 (KRT20) stains the villi but also extends to the crypt epithelium; thus, this marker cannot be used to define the villus-crypt border. The staining of glucose transporter 2 (GLUT2) resembles that of KRT-20, as it also extends to the crypt epithelium. In apolipoprotein A4 (APOA4) staining, the villus epithelium was strongly stained, while the crypt epithelium remained negative. Both cytochrome P450 3A4 (CYP3A4) and intestinal fatty-acid binding protein (I-FABP) looked promising in healthy control specimens but in damaged samples CYP3A4 also stained crypt cells and I-FABP then again disappeared almost completely from the sample. Magnification 200x, hematoxylin counterstain.

**Figure 3:**
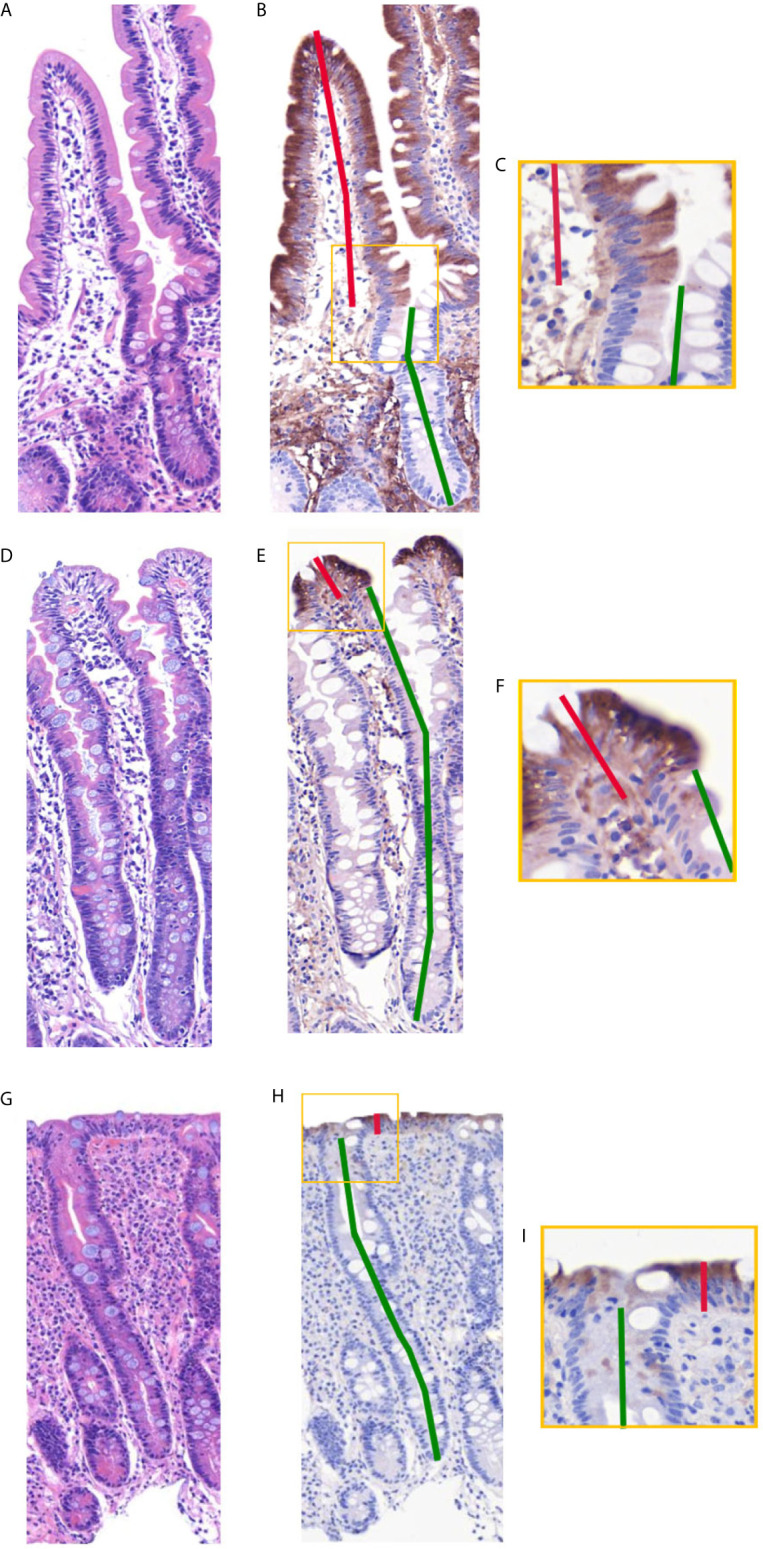
Side-by-side comparison of duodenal specimens by traditional hematoxylin-eosin (H&E) and apolipoprotein A4 (APOA4) after restaining. **(A, D, G)** depict standard H&E-stained specimens, and panels **(B, E, H)** depict APOA4-stained specimens. The border between villi and crypts is clearly visible in APOA4-stained specimens, as also seen in closeups **(C, F, I)**. **(D–F)** and **(G–I)** present the common pitfall of a long crypt basin. This long crypt basin can be misread as villi in H&E staining **(D, G)**, but with the aid of APOA4 staining **(E, H)**, it is clear that the crypt extends up close to the lumen, rendering the histological diagnosis of total villous atrophy in both cases. The VH : CrD ratios in the samples are 1.5 in **(A–C)**, 0.1 in **(D–F)**, and 0.1 in **(G–I)**. Magnification 200x, hematoxylin and eosin counterstaining in **(B, E, H)**.

In the comparison between APOA4, Ki-67, GLUT2, KRT20, CYP3A4 and I-FABP, APOA4 was chosen as the best candidate for further study (**Figure 2**). APOA4 labeling was specific for villus enterocytes and did not stain the crypt epithelium. The experiments with Ki-67, GLUT2, KRT20, CYP3A4 and I-FABP stainings yielded unsatisfactory results in demonstrating the villus-crypt border accurately (**Figure 2**). The Ki67-labeled proliferating crypt epithelium cells did not extend to the crypt-villus junction, rendering Ki67 staining unsuitable for our approach. In addition, proliferating IELs are also Ki-67 positive, interfering with the analysis. GLUT2 and KRT20 were stained in the villi, but the staining continued to some extent to the crypt. The CYP3A4 and I-FABP stainings were promising in healthy mucosa, however, in the damaged mucosa the stainings did not represent the villus-crypt junction. In the APOA4 staining (**Figure 2**), the villus-crypt border aligned properly, and the positively stained villus epithelium stopped abruptly, making the placement of the borderline easy. In damaged mucosa, long crypt basins can be misread as villi in H&E staining (**Figures 3D, G**), but with the aid of APOA4 staining (**Figures 3E, H**), it can be seen that the crypt extends up close to the lumen, resulting in a histological diagnosis of total villous atrophy in both cases.

**Figure 4:**
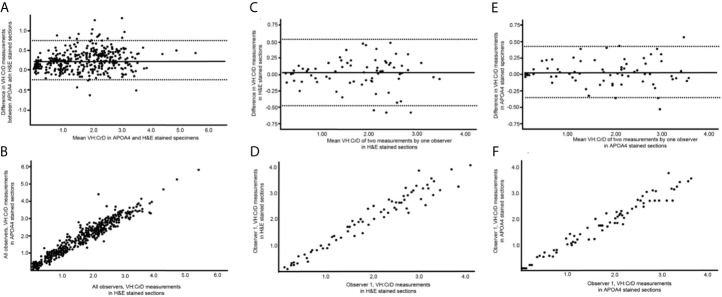
Graphical illustrations of the reliability and reproducibility of the villus height crypt depth ratio (VH : CrD) in hematoxylin-eosin (H&E)- and apolipoprotein A4 (APOA4)-stained specimens. **(A, C)** show Bland-Altman plots, and **(B, D)** present the regression analyses for intraobserver analyses of VH : CrD in H&E and APOA4 staining, respectively. **(E, F)** show Bland-Altman plots and regression analysis between H&E and APOA4 staining in the VH : CrD measurements of all observers. The solid lines in **(A, B)** indicate the mean difference between the measurements, and the dashed lines correspond to the 95% limits of agreement.

There were 69 readable samples with at least 3 villus-crypt units for the intraobserver analysis among the 74 evaluated samples. Observers 2, 3, 4 and 5 identified 65, 64, 57, and 61 readable samples, respectively. Five samples were unreadable to all; in all others, at least two observers measured at least 3 villus-crypt units on the sample. The mean villus heights, crypt depths and VH : CrD values in H&E-stained and APOA4-stained specimens are presented in **Table 1**. APOA4 staining made the assessment of the villus-crypt border easier in difficult cases by marking an objective villus-crypt junction site (**Figure 3**, **Supplementary Figure 1**). There was constant excellent agreement among all observers between H&E and APOA4 staining (**Table 1**). When comparing VH : CrD measurements by all observers between the methods, the mean difference was 0.227 with limits of agreement from −0.302 to 0.756 (**Figure 4A**); the standard deviation (SD) was 0.529. There was a significant mean difference between the methods in villus height, crypt depth and VH : CrD measurements (**Table 1**). Logistic regression analysis (**Figure 4B**) indicated the following conversion equation between the two staining methods: VH : CrD in H&E  = 0.2 + 1.01 * VH : CrD in APOA4.

**Table 1:** Comparison of villous height, crypt depth and villous height crypt depth ratio (VH : CrD) between hematoxylin-eosin (H&E) and apolipoprotein A4 (APOA4) stained specimens.

	Mean (range) in H&E, µm	Mean (range) in APOA4, µm	Mean difference	Correlation co-efficient
**Villous height**				
Observer 1^*^	399 (23–790)	386 (19–793)	12.7^**^	0.988^**^
*Observer 2^†^*	381 (38–755)	366 (23–741)	14.6^**^	0.987^**^
*Observer 3^‡^*	377 (38–643)	350 (29–649)	27.2^**^	0.981^**^
*Observer 4^§^*	401 (43–668)	384 (22–655)	17.5^**^	0.978^**^
*Observer 5^¶^*	399 (35–685)	371 (23–675)	27.5^**^	0.972^**^
***Total^#^***	**391 (23–790)**	**372 (19–793)**	19.7^**^	0.981^**^
**Crypth dept**				
Observer 1^*^	238 (121–458)	255 (130–525)	-16.7^**^	0.941^**^
*Observer 2^†^*	237 (130–447)	257 (137–558)	-20.2^**^	0.937^**^
*Observer 3^‡^*	231 (122–476)	258 (129–529)	-27.2^**^	0.947^**^
*Observer 4^§^*	237 (121–466)	255 (142–520)	-18.0^**^	0.925^**^
*Observer 5^¶^*	235 (131–534)	257 (143–522)	-22.5^**^	0.906^**^
***Total^#^***	**236 (121–534)**	**257 (129–558)**	-21.0^**^	0.928^**^
**VH : CrD**				
Observer 1^*^	2.02 (0.10-4.11)	1.80 (0.10-3.84)	0.221^**^	0.979^**^
*Observer 2^†^*	1.90 (0.09-5.83)	1.73 (0.05-5.42)	0.167^**^	0.977^**^
*Observer 3^‡^*	1.93 (0.09-4.67)	1.63 (0.08-4.27)	0.300^**^	0.968^**^
*Observer 4^§^*	2.00 (0.09-4.41)	1.76 (0.06-3.91)	0.237^**^	0.914^**^
*Observer 5^¶^*	2.00 (0.07-5.21)	1.73 (0.05-4.72)	0.273^**^	0.962^**^
***Total^#^***	**1.97 (0.07-5.83)**	**1.73 (0.05-5.42)**	0.233^**^	0.962^**^

*n=69, ^†^n=65, ^‡^n=64, ^§^n=57, ^¶^n=61, ^#^n=316, **p < 0.001. Bolded values represent the average value from the measurements of all observers together.

Our main purpose was to study the reliability and reproducibility of VH : CrD measurements when using APOA4 IHC when compared with traditional H&E. For this comparison, we analyzed the same biopsy sections after destaining and restaining with APOA4. In the intraobserver VH : CrD analyses, the mean differences in the two measurement series were less than 0.1, ensuring that there was no systematic measuring error between the measurement series. In the intraobserver Bland-Altman plots (**Figures 4C, E**), the 95% limits of agreement ranged from -0.476 to 0.528 for H&E and -0.356 to 0.420 for APOA4. The 2xSD error range of the measurements was 0.528 for H&E and 0.388 for APOA4 staining of the same tissue sections. The intraobserver logistic regression analyses are shown in **Figures 4D, F**, and the ICCs are shown in **Table 2**. In the interobserver analyses, all VH : CrDs by all observers showed smaller SDs and better ICCs in APOA4 than in H&E staining (**Table 2**). The average error ranges in interobserver analyses were 0.519 in H&E and 0.432 in APOA4. The mean differences in the interobserver analyses, indicating the observer dependency of the measurements, ranged from 0.074 to 0.219 for H&E staining and from 0.067 to 0.251 for APOA4 staining (**Table 2**).

**Table 2:** Bland-Altman statistics with absolute values and intraclass correlation coefficients (ICC) for analysing agreement and repeatability in small-bowel mucosal villus height crypt depth ratio (VH : CrD).

	Mean difference (95% CI)	Standard deviation	ICC
**VH : CrD in H&E**			
Intraobserver*	0.026 (-0.036 to 0.087)	0.256	0.971
*Interobserver, Observer 2^†^*	0.099 (-0.026 to 0.223)	0.491	0.897
*Interobserver, Observer 3^‡^*	0.074 (-0.040 to 0.188)	0.442	0.914
*Interobserver, Observer 4^§^*	0.219 (0.078 to 0.362)	0.534	0.862
*Interobserver, Observer 5^¶^*	0.127 (-0.032 to 0.286)	0.608	0.827
**VH : CrD in APOA4**			
Intraobserver^*^	0.032 (-0.015 to 0.080)	0.198	0.980
*Interobserver, Observer 2^†^*	0.067 (-0.049 to 0.182)	0.445	0.905
*Interobserver, Observer 3^‡^*	0.172 (0.080 to 0.264)	0.357	0.937
*Interobserver, Observer 4^§^*	0.251 (0.139 to 0.364)	0.424	0.900
*Interobserver, Observer 5^¶^*	0.205 (0.074 to 0.336)	0.503	0.869

CI, confidence interval. *n=69; ^†^n=65; ^‡^n=64; ^§^n=57; ^¶^n=61.

## Discussion

The present study shows that immunohistochemical staining of APOA4 defines the villus-crypt border by separating the differentiated villus epithelium and proliferating crypt epithelium. The villus-to-crypt ratios were analyzed with quantitative morphometry according to our standard operating procedure used in previous publications and gluten challenge trials (4, 5, 11, 12, 14, 24, 29). The correlation coefficients and Bland-Altman analyses showed excellent agreement between the results from APOA4 staining and the standard and validated H&E staining. Hence, APOA4 staining can be used as an objective marker of the villus-crypt border in analysis of the duodenal mucosal architecture in celiac disease. The addition of APOA4 staining to the immunohistochemistry workout is relatively easy because CD3 IHC staining of IELs is included routinely in translational celiac disease studies and clinical trials (2, 11, 12, 14, 29).

We adopted APOA4 as an immunohistochemical marker of the villus epithelium. Its function has not been linked to celiac disease so far. It is a lipid-binding 46 kD glycoprotein that is almost exclusively synthesized in the absorptive enterocytes of the small intestine, packaged into chylomicrons, and secreted into intestinal lymph during fat absorption (30). APOA4 is involved in several physiological processes, such as lipid absorption and metabolism (31), antiatherosclerosis (32), anti-inflammatory agents (33), glucose homeostasis, and food intake (34). Previously, we showed that the mRNA expression levels of APOA4 are decreased in untreated celiac disease and after gluten challenge (4, 24). The decrease in APOA4 in the gluten-induced duodenal lesion in celiac disease showing villous atrophy and crypt hyperplasia is the logical result of the loss of mature absorptive villus epithelium, as shown in **Figures 2** and **3**.

The distinction of the border between villi and crypts is of utmost importance in assessing celiac disease biopsy specimens (2, 11, 20). Currently, the placement of this border is debatable and lacks scientific rationale in traditional analyses based on H&E staining. Ground truth differentiation between villi and crypt epithelium can be done only by transmission electron microscopy (20), but because microvilli are not visible in H&E staining, researchers and pathologists use subjective pattern recognition to define the villus-crypt border according to the notch or a plateau usually seen at the border (**Figures 2A–C**). However, problems arise in celiac disease biopsies showing crypt hyperplasia in addition to villous atrophy. The long crypt collars or large open “basins” in a totally flat mucosal lesion (Marsh III) can be misinterpreted as villi (20, 35). In these samples, the notch or plateau was missing, and it was difficult to place the villus-crypt border (**Figure 2D**). In such instances, APOA4 staining provides a new possibility to define the villus-crypt border objectively and accurately (**Figures 2E, I**). The VH : CrD values were lower for APOA4 staining by a factor of approximately 0.2, indicating that the villus-crypt border appears somewhat lower with APOA4 staining than with H&E staining (**Table 1**, **Figure 4A**). For example, a VH : CrD value of 2.0, which is considered a borderline value for healed mucosa in celiac patients on a gluten-free diet (2, 5, 36), would equal 1.8 in APOA4 staining. We believe that with APOA4 staining, the reader has more confidence to place the border correctly and somewhat higher than in H&E staining, which might reflect the epithelial border better than in traditional H&E staining (see **Figure 2D–I**). Hence, APOA4 staining can be particularly helpful in borderline cases in which incorrect diagnoses may occur (15). The addition of eosin to the APOA4 staining procedure helps to identify the base of the crypt by staining the Paneth cells and thus ensuring that the entire crypt is considered.

In our study, APOA4 staining improved the reliability and reproducibility of VH : CrD measurements in celiac disease biopsy specimens in comparison to traditional H&E-stained sections. The standard deviations were smaller, and the ICCs were better both in intraobserver and in all interobserver analyses in APOA4-stained sections. Low interobserver agreement has been a concern in celiac disease histology (11, 15–17, 19). In the work by Werkstetter et al., two pathologists reviewed the same duodenal samples in a blinded manner, and in 11% of cases, the histological diagnosis changed from normal to celiac disease or vice versa (see Supplementary Table S21 in the article by Werkstetter et al.) (15). To remove such drastic problems in reading the samples, objective reading tools are needed for analysis of the duodenal mucosa to obtain reliable and reproducible results (2). Additionally, the use of the same reader or readers is essential to minimize variation in measurements, as interobserver analyses have significantly higher error ranges than intraobserver analyses, as also shown in this study. Hence, in our standard operating procedure, the sample is read by two or three blinded main readers, and then, in controversial results, a senior pathologist can counter this pitfall in second-opinion slide reading (11). The advantages of APOA4 in reliability and reproducibility is especially useful in pharmacological intervention studies in which small but significant changes in VH : CrD need to be observed (14, 29). In gluten challenge studies or when assessing the effect of a gluten-free diet with APOA4 staining, a conservative cutoff of a clinically relevant difference of 0.4 between the paired measurements was derived from the intraobserver Bland-Altman analysis.

When searching for a suitable immunohistochemical marker, we evaluated several candidate markers shown to be specific for either villus or crypt epithelium. Of these, the proliferating Ki-67-positive cells are increased due to the compensatory proliferation of epithelial cells in the duodenal crypts. The mRNA levels of Ki-67 predict mucosal damage well, as shown in a previous study (24, 37). The gene expression of GLUT2 and KRT20 showed significant reactions to gluten challenge in our previous study and was thus interesting prospects for the staining of the villus-crypt border (4). However, Ki-67, GLUT2 and KRT20 IHC staining was not optimal for defining the villus-crypt border by IHC, as shown in **Figure 2**. CYP3A4 and I-FABP have previously shown promise as blood biomarkers in predicting duodenal damage in celiac disease (38, 39). Both also looked promising as markers of villus-crypt border in healthy control samples, however, in damaged duodenal mucosa CYP3A4 also stained the crypt cells and I-FABP was almost completely absent from epithelium making these stainings unsuitable for this study. Based on epithelial differentiation, a direct microvillus marker, such as villin or CD10 (40), could be useful in our approach. However, villin and CD10 also stain the immature (forming) microvilli present in the crypt cells, making these cells unsuitable for VH : CrD assessments (20).

Previous studies have shown that the secretion of APOA4 into lymph is stimulated by lipid absorption (41) and that the plasma APOA4 correlates positively with plasma triglycerides (42). In addition, mRNA levels of APOA4 have been found to respond in a tissue specific-manner to a number of factors such as estrogen, thyroid hormone, corticosteroid and insulin (43, 44). These factors could also potentially affect APOA4 staining in duodenum, however, the effect of these on APOA4 staining in the small bowel has not been studied. A common pitfall in any IHC staining is also too weak staining intensity. In this study, the APOA4 staining was strong and had a clear cut-off for villus-crypt junction in wide variety of duodenal injuries (**Figure 3**, **Supplementary Figure 1**). Also, previously a decrease in mRNA expression of APOA4 has been shown to follow duodenal injury (4, 45). These findings provide support that the staining is not significantly affected by confounding factors. We titrated the antibody reagent carefully and obtained a nearly identical staining pattern with another APOA4 antibody (clone G-8). Despite potential pitfalls, APOA4 staining seemed to work in this controlled environment quite well.

APOA4 staining defines the villus crypt border accurately and objectively. The reliability and reproducibility of APOA4 is better than that of traditional H&E-stained specimens. APOA4 staining is easy to perform and allows coordinated analysis of the duodenal mucosa in celiac disease that has not been possible before. These findings are important for analyzing subtle differences in celiac disease biopsies.

## Data Availability Statement

The raw data supporting the conclusions of this article will be made available by the authors, without undue reservation.

## Ethics Statement

The studies involving human participants were reviewed and approved by Regional Ethics Committee of the Expert Responsibility area of Tampere University Hospital. The patients/participants provided their written informed consent to participate in this study.

## Author Contributions

JT, KV, MM, and JI: study concept and design. JT, AV, JS, KS, and JI: acquisition of data. JT, MM, and JI: analysis and interpretation of data. JT, MM, and JI: drafting of the manuscript. All authors were involved in the critical revision of the manuscript for important intellectual content. All authors contributed to the article and approved the submitted version.

## Funding

This work was supported by grants from the Competitive State Research Financing of the Expert Responsibility Area of Kuopio University Hospital (B2009), Competitive State Research Financing of the Expert Responsibility Area of Tampere University Hospital (9X035 and 9AB048), Emil Aaltonen Foundation and the Finnish Medical Foundation.

## Conflict of Interest

JI is the chief execute officer of Jilab Inc. that produces diagnostic laboratory services for small-bowel diseases and a wide variety of cancers. JS and KS are employees of Jilab Inc. AV is employed by Fimlab Laboratories Inc.

The remaining authors declare that the research was conducted in the absence of any commercial or financial relationships that could be construed as a potential conflict of interest.

The handling editor declared a past co-authorship with the authors JT, MM, and JI.

## Publisher’s Note

All claims expressed in this article are solely those of the authors and do not necessarily represent those of their affiliated organizations, or those of the publisher, the editors and the reviewers. Any product that may be evaluated in this article, or claim that may be made by its manufacturer, is not guaranteed or endorsed by the publisher.
