# Supplementary figures and images for: Apolipoprotein A4 Defines the Villus-Crypt Border in Duodenal Specimens for Celiac Disease Morphometry
Source: Front Immunol. 2021 Jul 29;12:713854. doi: 10.3389/fimmu.2021.713854 (PMC8358775; doi:10.3389/fimmu.2021.713854)

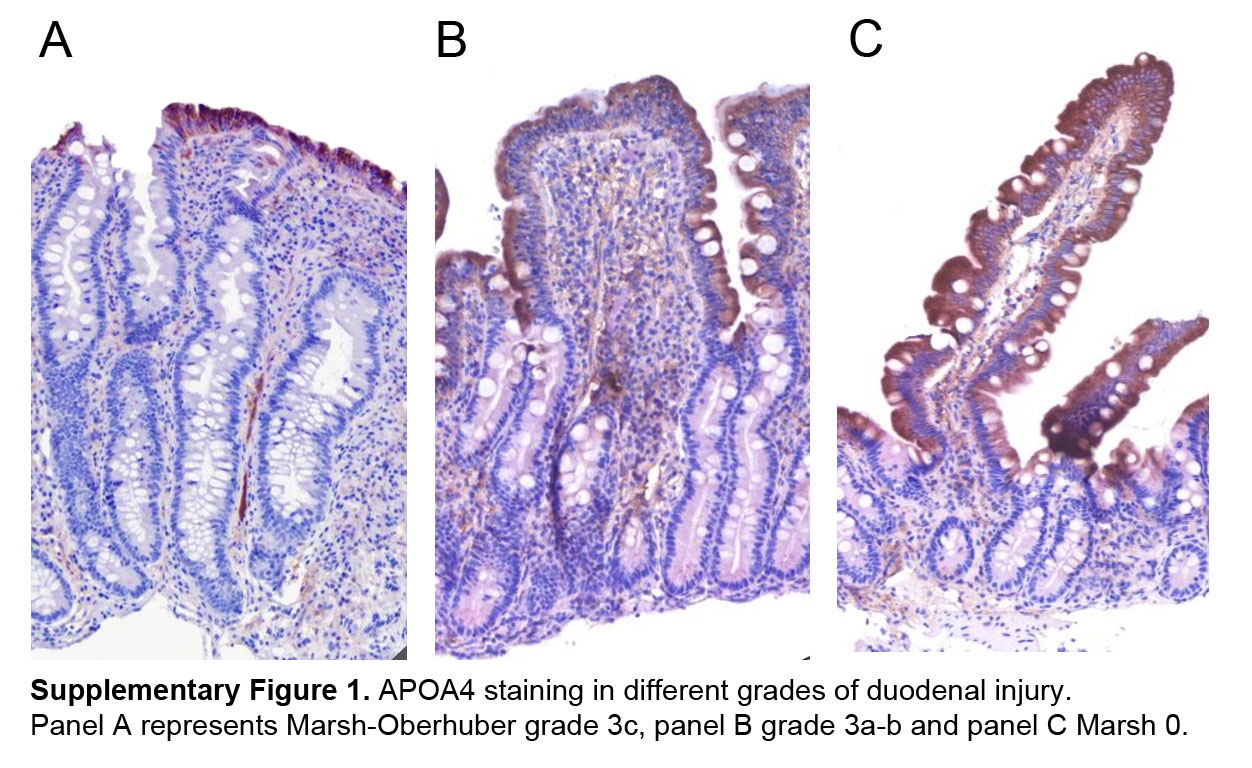

Supplement: Supplementary file 1 [file Image_1.jpeg]
