# Supplementary material for: Apolipoprotein A4 Defines the Villus-Crypt Border in Duodenal Specimens for Celiac Disease Morphometry
Source: Front Immunol. 2021 Jul 29;12:713854. doi: 10.3389/fimmu.2021.713854 (PMC8358775; doi:10.3389/fimmu.2021.713854)
Supplement: Supplementary file 2 [file Table_1.docx]

**Supplementary Table 1.** The antibodies used in the immunohistochemical stainings and their respective dilutions.

| Protein | Antibody clone | Product ID | Dilution | Vendor |
| --- | --- | --- | --- | --- |
| Apolipoprotein A4 | Rabbit polyclonal | HPA001352 | 1:3000 | Sigma Atlas antibodies |
| Apolipoprotein A4 | mouse monoclonal | sc-374543 | 1:3000 | Santa Cruz Biotechnology |
| Ki-67 | MIB-1 | M7240 | 1:100 | Dako-Agilent |
| Glucose transporter 2 | Rabbit polyclonal | HPA028997 | 1:300 | Sigma Atlas antibodies |
| Keratin 20 | Ks20.8 | BSB5389 | 1:3000 | Bio SB |
| Cytochrome P450 3A4 | mouse monoclonal | sc-53850 | 1:1000 | Santa Cruz Biotechnology |
| Intestinal fatty-acid binding protein | mouse monoclonal | CPTC-FABP2-1-s | 1:500 | Developmental Studies Hybridoma Bank |
